# Levels of Adiponectin Expression in Peri-Renal and Subcutaneous Adipose Tissue and Its Determinants in Human Biopsied Samples

**DOI:** 10.3389/fendo.2019.00897

**Published:** 2020-02-06

**Authors:** Gulinu Maimaituxun, Daiju Fukuda, Hirofumi Izaki, Yoichiro Hirata, Hiro-omi Kanayama, Hiroaki Masuzaki, Masataka Sata, Michio Shimabukuro

**Affiliations:** ^1^Department of Diabetes, Endocrinology and Metabolism, School of Medicine, Fukushima Medical University, Fukushima, Japan; ^2^Department of Cardiovascular Medicine, Tokushima University Graduate School of Biomedical Sciences, Tokushima, Japan; ^3^Department of Cardio-Diabetes Medicine, Tokushima University Graduate School of Biomedical Sciences, Tokushima, Japan; ^4^Department of Urology, Tokushima Prefectural Central Hospital, Tokushima, Japan; ^5^Department of Pediatrics, Graduate School of Medicine, The University of Tokyo, Tokyo, Japan; ^6^Department of Urology, Tokushima University Graduate School of Biomedical Sciences, Tokushima, Japan; ^7^Division of Endocrinology, Diabetes and Metabolism, Hematology, Rheumatology (Second Department of Internal Medicine), Graduate School of Medicine, University of the Ryukyus, Okinawa, Japan

**Keywords:** adiponectin, kidney, visceral abdominal fat, adipose accumulation, renal fat

## Abstract

**Background:** The interactions of adipose tissue with the kidney are hypothesized to affect kidney function. Also, excessive peri-renal fat may increase the risk of cardiometabolic risk. However, the role(s) of peri-renal fat adipocytokine has never been evaluated.

**Objectives:** To elucidate levels of adiponectin expression in peri-renal and subcutaneous adipose tissue and its determinants in human biopsied samples.

**Methods:** A pair of subcutaneous and perirenal fat tissue samples were collected from 80 patients (men: 54; women: 26) who underwent urological operations. Subcutaneous adipose tissue (SAT) area, visceral adipose tissue (VAT) area and peri-renal adipose tissue (RAT) volume were quantified on abdominal computed tomography. Cytokine/adipocytokine expression was evaluated by real-time semi-quantitative polymerase chain reaction (qPCR). Probability was considered significant if *P* < 0.05.

**Results:** Current study evaluated determinants of plasma adiponectin levels and expression levels of adiponectin in SAT and RAT in human samples. We found that: first, plasma adiponectin levels were correlated with VAT area but not with BMI, waist circumference, SAT area, and RAT volume; second, expression levels of adiponectin in SAT were correlated with BMI, waist circumference, and SAT area but not with VAT area and RAT volume; and third, expression levels of adiponectin in RAT were correlated with all adiposity indices including BMI, waist circumference, SAT area, VAT area, and RAT volume.

**Conclusion:** This study evaluated levels of adiponectin expression in RAT and SAT and its determinants in patients who underwent urological operation. Levels of adiponectin mRNA in RAT were negatively correlated with remote fat mass in SAT and VAT and also with local fat mass in RAT, while level of adiponectin in SAT was not correlated with RAT volume. Further studies are warranted to evaluate roles of peri-renal fat mass accumulation and its pathophysiological machineries.

## Introduction

Adiponectin is a circulating protein exclusively produced from adipocytes and shows unique insulin-sensitizing, anti-inflammatory, and anti-apoptotic properties (1). Lower plasma levels of adiponectin are associated with prevalence and incidence of type 2 diabetes and cardiovascular disease (CVD), and higher levels of this molecule are associated with protection from cardiometabolic risk (2–4). Therefore, regulatory mechanisms of circulating adiponectin levels have been pursued vigorously, but it has been yet unclarified (5–8). It is known that protein production or gene expression of various adipocytokines is regulated in a depot-specific manner, e.g., subcutaneous adipose tissue (SAT) vs. visceral adipose tissue (VAT) (9–12). Central obesity and visceral fat obesity, which can be assumed by an increased VAT volume, have been shown to be associated with lower plasma levels of adiponectin (2–4). Thus, one can assume that a decrease in protein production or gene expression of adiponectin in VAT could explain hypoadiponectinemia at least partially.

Emerging evidence suggests that ectopic fat deposition, including hepatic, muscle, and cardiovascular fat, can contribute to increased CVD and cardiometabolic risk (13–15). Likewise, the interactions of adipose tissue with the kidney are also hypothesized to affect kidney function (16, 17). Epidemiological studies suggest that excessive perirenal fat increases the risk of cardiometabolic risk (18), hypertension (19), and CVD (20). Consequently, it can be assumed that RAT is associated with kidney functions and/or cardiometabolic risk via paracrine signaling of adipocytokine (17). However, such role(s) of RAT adipocytokine has never been evaluated.

In this study, we evaluated levels of adiponectin expression in RAT and its determinants as compared to that of SAT in human biopsied samples from patients who underwent urological operation.

## Methods

### Subjects

The protocol of this study was approved by the institutional review boards of the University of Tokushima Hospital. All subjects gave their written informed consent before beginning the study. A pair of SAT and RAT samples were consecutively collected from 80 patients (Male: 54; Female: 26) who underwent urological operations at the Department of Urology, The University of Tokushima Graduate School, between April 2011 and July 2012. Almost all patients were operated for removal of early cancer or benign tumors (see details in Table 1). Before the operations, pair samples ~100 mg were obtained from SAT in the abdominal wall and from RAT in perirenal tumor-free areas. Clinical parameters were obtained from electronic medical records. Hypertension was defined as blood pressure ≥140/90 mmHg or the current use of antihypertensive medication(s). Diabetes was defined as HbA_1c_ ≥6.5%, fasting plasma glucose levels >126 mg/dl, or the current use of antidiabetic medications. Dyslipidemia was defined as a total serum cholesterol level ≥220 mg/dl, LDL cholesterol level ≥140 mg/dl, serum triglyceride level >150 mg/dl, and a serum HDL cholesterol level of <40 mg/dl, and/or current use of anti-hyperlipidemia medications. Smoking was defined as the patient being a past or current smoker; non-smoking was defined as a patient who had never smoked.

**Table 1:** Clinical characteristics of studied subjects.

**Demographic data**	
Number	80
Age, years	62 ± 14
Men, n (%)	54 (79%)
Body weight, kg	61 ± 10
Body mass index, kg/m^2^	23.5 ± 3.5
**Metabolic disease**	
Overweight (BMI ≥25), n (%)	26 (33%)
Smoking, n (%)	29 (36%)
Hypertension, n (%)	43 (54%)
T2DM, n (%)	23 (29%)
Hyperlipidemia, n (%)	44 (55%)
**Urological disease**	
Renal cell carcinoma, n (%)	28 (35%)
Prostate cancer, n (%)	16 (20%)
Primary aldosteronism, n (%)	2 (2.5%)
Bladder cancer, n (%)	5 (6%)
Renal pelvis carcinoma, n (%)	6 (7.5%)
Ureteral carcinoma, n (%)	4 (5%)
Healthy donor, n (%)	2 (2.5%)
Pheochromocytoma, n (%)	1 (1.3%)
Adrenal tumor, n (%)	6 (7.5%)
**Hemodynamic data**	
Systolic blood pressure, mmHg	124 ± 14
Diastolic blood pressure, mmHg	74 ± 11
Heart rate, beats/min	74 ± 14
**Laboratory data**	
GOT, IU/L	22 ± 9
GPT, IU/L	20 ± 13
ɤGTP, IU/L	36 ± 35
Uric acid, mg/dL	5.4 ± 1.4
Creatinine, mg/dL	0.8 ± 0.2
eGFR ml/min/1.73m^2^	62 ± 20
Adiponectin, μg/mL	4.0 ± 2.9
Insulin, mU/L	4.4 ± 3.2
HOMA-IR	1.2 ± 0.9
**Adiposity data**	
Waist circumference, cm	87.0 ± 10.0
Subcutaneous adipose tissue area, cm^2^	142 ± 71
Visceral adipose tissue area, cm^2^	106 ± 55
Renal adipose tissue volume, cm^3^	80 ± 44
**Medication**	
Angiotensin-converting-enzyme inhibitor, n (%)	4 (5%)
Angiotensin II receptor blocker, n (%)	11 (14%)
Calcium channel blocker, n (%)	31 (39%)
Statin, n (%)	6 (7.5%)

*Data are expressed as mean ± SD or n (%)*.

### Adipose Tissue Quantification by Computed Tomography

CT images were transferred to an offline workstation (Synapse Vincent, ver. 4.4, Fuji Film, Tokyo, Japan). SAT area, VAT area, and waist circumference were measured in umbilical level on non-contrast computed tomography (CT) as described (21, 22). The peri-renal fat area surrounding the kidney (RAT area) was determined on axial views by placing region of interest (ROI) on the renal fascia with modifications (19), and RAT area of each slice was summed and multiplied by the slice thickness and number of slices to determine RAT volume. Adipose tissue was highlighted and computed using an attenuation range of −190 to −30 Hounsfield Units.

### Cytokine/Adipocytokine Measurements

Cytokine/adipocytokine expression was evaluated as described (11, 23). Briefly, RNA was extracted from adipose tissue samples by using an RNeasy Lipid Tissue Mini kit (QIAGEN KK, Tokyo). Cytokine/adipocytokine expression was evaluated by real-time quantitative polymerase chain reaction (qPCR) with a TaqMan Gold RT-PCR kit and PRISM 7500 Sequence Detection System (Applied Biosystems Applied Biosystems Japan, Tokyo). The primer and probe sets used for quantitative real-time PCR analysis are shown previously (11). Primers were purchased from Takara Bio (Kyoto, Japan). Data were quantified using the DDCT method.

### Biochemical Measurements

Venous blood samples were obtained in tubes without anticoagulant or with EDTA sodium (1 mg/ml). Plasma was immediately separated by centrifugation at 3,000 rpm at 4°C for 10 min and serum by centrifugation at 1,000 rpm at room temperature for 10 min. Samples were stored at −80°C until assayed. Routine chemical methods were used to determine plasma concentration of glucose and serum concentrations of LDL- and HDL-cholesterol, triglycerides, creatinine, and glucose. Glycated hemoglobin (hemoglobin A1c [HbA1c]) was measured by high-performance liquid chromatography and insulin by chemiluminescent enzyme immunoassay. High molecular weight (HMW) adiponectin concentration was measured in plasma by a sandwich ELISA (Otsuka Pharmaceuticals, Tokyo, Japan) (23). We calculated estimated glomerular filtration rate (eGFR) using the Japanese formula for GFR estimation, i.e., eGFR (ml/min/1.73 m^2^) = 194 × serum creatinine (mg/dl) ^−1.094^ × age (years)^−0.287^ as a marker of renal function (24).

### Statistical Analysis

Variables are expressed as means (± standard deviations) or frequencies. Correlations between variables were determined using Spearman's correlation coefficient tests. The impact of independent parameters on levels of adiponectin in plasma, SAT, and RAT (dependent parameters) was evaluated by multivariate regression models. Possible independent parameters were included to models in a hierarchical fashion. For model of plasma adiponectin, known clinical parameters such as age, gender, current smoking, hyperlipidemia, hypertension, and type 2 diabetes and BMI were included in model 1. In models 2 to 6, SAT area, VAT area, RAT volume, SAT adiponectin, and RAT adiponectin were replaced for BMI, in addition to the parameters in model 1. For model of adiponectin in SAT and RAT, models 1–4 were considered as in plasma adiponectin. For all tests, statistical significance was set at *P* < 0.05. All statistical analyses were performed using SPSS 21.0 for Windows (SPSS, Chicago, IL).

## Results

### Clinical Characteristics of Studied Patients

Characteristics of the patients enrolled in this study are shown in Table 1. Patients with average age of 62 years showed a body mass index of 23.5 ± 3.5 kg/m^2^, among which 33% (*n* = 26) were overweight and 3% (*n* = 2) were obese (BMI ≥ 30). Among 80 patients, 64 had early malignancy and 18 had non-malignant diseases. All patients were not critically ill and did not have severe kidney dysfunction and had underwent operations successfully without complications.

### Univariate and Multivariate Regression Analysis to Estimate Adiponectin in Plasma, Subcutaneous Adipose Tissue, and Peri-Renal Adipose Tissue

**Figure 1:**
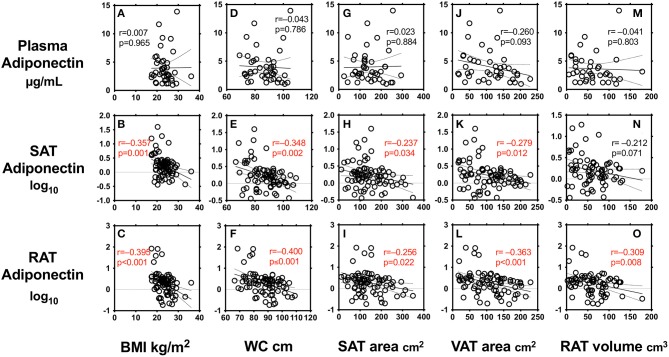
Correlations between body mass index (BMI) **(A–C)**, waist circumference (WC) **(D–F)**, subcutaneous adipose tissue (SAT) area **(G–I)**, visceral adipose tissue (VAT) area **(J–L)**, peri-renal adipose tissue (RAT) volume **(M–O)**, and plasma adiponectin levels and expression levels in SAT and RAT r: Pearson's simple regression analysis, p: *p*-values.

We first performed simple regression analysis to estimate adiponectin in plasma, SAT, and RAT with adiposity markers including BMI, waist circumference, SAT area, VAT area, and RAT volume (Figure 1). BMI, waist circumference, SAT area, and VAT area were negatively correlated with SAT and RAT adiponectin, but not with plasma adiponectin. RAT volume was negatively correlated with RAT adiponectin, but not with plasma adiponectin and SAT adiponectin. Multivariate regression analysis showed that VAT area, but not SAT area nor RAT volume, was a determinant for plasma adiponectin levels after corrected for known confounding factors such as age, gender, hyperlipidemia, hypertension, type 2 diabetes, and smoking (Table 2). For adiponectin expression levels in SAT, SAT area, but not VAT area nor RAT volume, was a determinant. SAT and VAT area and RAT volume were all determinants for adiponectin expression levels in RAT.

**Table 2:** Univariate and multiple regression analysis to estimate adiponectin in plasma, subcutaneous adipose tissue (SAT) and renal adipose tissue (RAT).

	**Multiple regression analysis**													
	**Univariate regression analysis**		**Model 1**		**Model 2**		**Model 3**		**Model 4**		**Model 5**		**Model 6**	
	**Standarized β**	***P*-value**	**Standarized β**	***P*-value**	**Standarized β**	***P*-value**	**Standarized β**	***P*-value**	**Standarized β**	***P*-value**	**Standarized β**	***P*-value**	**Standarized β**	***P*-value**
**PLASMA ADIPONECTIN**														
Age, years	0.246	0.112	0.417	0.023	0.428	0.019	0.521	0.004	0.460	0.054	0.403	0.039	0.414	0.026
Male gender, yes or no	−0.084	0.594	−0.226	0.227	−0.246	0.188	−0.092	0.617	−0.168	0.449	−0.208	0.308	−0.216	0.269
Current smoking status, yes or no	−0.084	0.611	0.026	0.885	0.012	0.946	−0.003	0.984	0.079	0.695	0.024	0.898	0.021	0.909
Hyperlipidemia, yes or no	−0.292	0.057	−0.334	0.059	−0.345	0.050	−0.296	0.072	−0.030	0.882	−0.319	0.092	−0.326	0.074
Hypertension, yes or no	−0.097	0.537	−0.173	0.336	−0.197	0.270	−0.137	0.411	−0.240	0.237	−0.170	0.352	−0.173	0.344
Type 2 diabetes mellitus, yes or no	0.108	0.491	0.060	0.731	0.062	0.720	0.010	0.950	0.043	0.821	0.073	0.696	0.054	0.764
Body mass index, kg/m^2^	0.007	0.964	−0.058	0.735	–	–	–	–	–	–	−0.034	0.863	−0.043	0.818
Subcutaneous adipose tissue area, cm^2^	0.023	0.884	–	–	−0.120	0.464	–	–	–	–	–	–	–	–
Visceral adipose tissue area, cm^2^	−0.260	0.093	–	–	–	–	−0.387	0.034	–	–	–	–	–	–
Renal adipose tissue volume, cm^3^	0.041	0.804	–	–	–	–	–	–	−0.169	0.416	–	–	–	–
Adiponectin_SAT (log)	0.184	0.238	–	–	–	–	–	–	–	–	0.051	0.813	–	–
Adiponectin_RAT (log)	0.142	0.363	–	–	–	–	–	–	–	–	–	–	0.040	0.826
**ADIPONECTIN IN SUBCUTANEOUS ADIPOSE TISSUE**														
Age, years	0.073	0.517	0.173	0.109	0.143	0.185	0.164	0.159	0.125	0.335	–	–	–	–
Male gender, yes or no	−0.276	0.013	−0.400	0.001	−0.478	<0.001	−0.357	0.005	−0.296	0.030	–	–	–	–
Current smoking status, yes or no	0.100	0.394	0.214	0.055	0.199	0.079	0.230	0.055	0.258	0.041	–	–	–	–
Hyperlipidemia, yes or no	0.156	0.166	0.016	0.878	0.015	0.886	0.034	0.765	0.128	0.293	–	–	–	–
Hypertension, yes or no	−0.255	0.044	−0.292	0.010	−0.328	0.004	−0.321	0.008	−0.332	0.010	–	–	–	–
Type 2 diabetes mellitus, yes or no	−0.300	0.007	−0.091	0.384	−0.109	0.296	−0.118	0.286	0.144	0.224	–	–	–	–
Body mass index, kg/m^2^	−0.356	0.001	−0.335	0.002	–	–	–	–	–	–	–	–	–	–
Subcutaneous adipose tissue area, cm^2^	−0.237	0.034	–	–	−0.327	0.003	–	–	–	–	–	–	–	–
Visceral adipose tissue area, cm^2^	−0.279	0.012	–	–	–	–	−0.153	0.204	–	–	–	–	–	–
Renal adipose tissue volume, cm^3^	−0.213	0.071	–	–	–	–	–	–	−0.055	0.674	–	–	–	–
**ADIPONECTIN IN RENAL ADIPOSE TISSUE**														
Age, years	−0.076	0.503	0.094	0.414	0.058	0.622	0.120	0.317	0.114	0.392	–	–	–	–
Male gender, yes or no	−0.189	0.092	−0.277	0.024	−0.343	0.009	−0.186	0.148	−0.124	0.368	–	–	–	–
Current smoking status, yes or no	0.182	0.12	0.250	0.037	0.246	0.049	0.242	0.050	0.289	0.025	–	–	–	–
Hyperlipidemia, yes or no	0.122	0.28	−0.008	0.941	−0.013	0.910	0.049	0.679	0.099	0.423	–	–	–	–
Hypertension, yes or no	−0.15	0.184	−0.166	0.163	−0.210	0.086	−0.167	0.172	−0.170	0.189	–	–	–	–
Type 2 diabetes mellitus, yes or no	−0.218	0.052	−0.065	0.560	−0.091	0.430	−0.078	0.494	−0.103	0.393	–	–	–	–
Body mass index, kg/m^2^	−0.395	0.001	−0.364	0.002	–	–	–	–	–	–	–	–	–	–
Subcutaneous adipose tissue area, cm^2^	−0.256	0.022	–	–	−0.277	0.019	–	–	–	–	–	–	–	–
Visceral adipose tissue area, cm^2^	−0.363	0.001	–	–	–	–	−0.331	0.009	–	–	–	–	–	–
Renal adipose tissue volume, cm^3^	−0.309	0.008	–	–	–	–	–	–	−0.270	0.045	–	–	–	–

### Association of Adiposity Indices With Levels of Adipocytokines in Subcutaneous Adipose Tissue and Peri-Renal Adipose Tissue

The correlations between adiposity indices with levels of adipocytokines in SAT and RAT are shown in Table 3. In SAT, increases in adiposity indices, except RAT volume, were negatively correlated with adiponectin levels. SAT area and RAT volume were positively correlated with CD68 levels. SAT adiponectin levels were positively correlated with IL18, TLR4, TLR9, and negatively with CD68 and NOX4. In RAT, all adiposity indices were negatively correlated with adiponectin levels. Waist circumference, VAT area, and RAT volume were positively correlated with CD68 levels. RAT adiponectin levels were positively correlated with TLR4 and TLR9 and negatively with NLRP3, TLR2, and CD68.

**Table 3:** Spearman correlation analysis between levels of adipocytokines expression in subcutaneous and renal adipose tissue and adiposity indices.

**Parameters**	**BMI, kg/m^2^**		**Waist circumference, cm**		**SAT area, cm**^****2****^		**VAT area, cm**^****2****^		**RAT volume, cm**^****3****^		**Adiponectin_SAT (log)**	
	*r*	*P*-value	*r*	*P*-value	*r*	*P*-value	*r*	*P*-value	*r*	*P*-value	*r*	*P*-value
**SUBCUTANEOUS ADIPOSE TISSUE (SAT)**												
IL1B_SAT (log)	−0.188	0.104	−0.106	0.362	−0.101	0.387	−0.160	0.167	−0.141	0.248	0.072	0.539
IL18_SAT (log)	0.083	0.466	0.045	0.694	0.081	0.473	0.151	0.182	0.071	0.553	0.269	0.016
NLRP3_SAT (log)	0.050	0.660	0.166	0.141	0.173	0.125	0.102	0.367	0.179	0.130	−0.186	0.098
Adiponectin_SAT (log)	−0.332	0.003	−0.350	0.001	−0.232	0.038	−0.280	0.012	−0.198	0.094	–	–
TLR2_SAT (log)	−0.107	0.346	−0.069	0.541	−0.041	0.721	−0.052	0.647	0.074	0.531	−0.029	0.800
TLR4_SAT (log)	−0.297	0.007	−0.339	0.002	−0.257	0.021	−0.334	0.002	−0.205	0.082	0.477	<0.001
TLR9_SAT (log)	−0.237	0.036	−0.224	0.047	−0.127	0.265	−0.268	0.017	−0.268	0.023	0.462	<0.001
HMGB1_SAT (log)	−0.099	0.382	−0.144	0.202	−0.099	0.381	0.018	0.876	0.110	0.354	0.112	0.322
CD68_SAT (log)	0.144	0.235	0.204	0.091	0.285	0.017	0.219	0.069	0.251	0.042	−0.324	0.006
NOX4_SAT (log)	0.135	0.341	0.143	0.313	0.013	0.925	0.070	0.622	0.232	0.112	−0.386	0.005
RENAL ADIPOSE TISSUE (RAT)												
IL1B_RAT (log)	−0.190	0.091	−0.203	0.071	−0.184	0.103	−0.065	0.564	−0.228	0.052	0.005	0.968
IL18_RAT (log)	−0.062	0.585	−0.078	0.492	−0.052	0.645	0.157	0.164	0.055	0.642	0.083	0.462
NLRP3_RAT (log)	0.125	0.270	0.100	0.379	0.069	0.542	0.205	0.068	0.259	0.027	−0.305	0.006
Adiponectin_RAT (log)	−0.397	<0.001	−0.360	0.001	−0.250	0.026	−0.374	0.001	−0.259	0.027	–	–
TLR2_RAT (log)	−0.020	0.860	−0.051	0.653	0.030	0.794	0.036	0.753	0.019	0.874	−0.307	0.006
TLR4_RAT (log)	−0.263	0.018	−0.306	0.006	−0.183	0.104	−0.204	0.070	−0.069	0.561	0.495	<0.001
TLR9_RAT (log)	−0.104	0.357	−0.047	0.681	0.042	0.713	−0.083	0.463	−0.059	0.620	0.289	0.009
HMGB1_RAT (log)	−0.114	0.313	−0.138	0.221	0.025	0.827	−0.065	0.567	0.098	0.409	0.126	0.265
CD68_RAT (log)	0.228	0.061	0.283	0.019	0.228	0.062	0.412	<0.001	0.490	<0.001	−0.348	0.004
NOX4_RAT (log)	0.038	0.794	0.094	0.514	0.158	0.268	−0.035	0.808	0.104	0.486	−0.111	0.437

### Association of Adiponectin and Adiposity Indices With Renal Function (Estimated Glomerular Filtration Rate)

**Figure 2:**
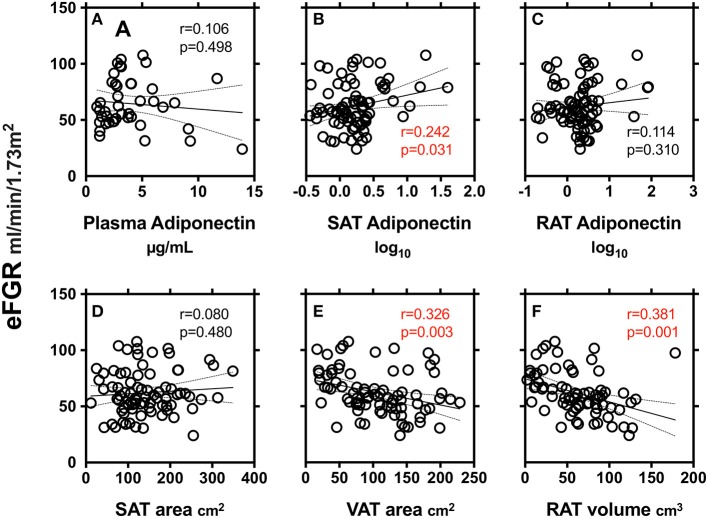
Correlations between markers of renal function (eGFR) and adiponectin in plasma **(A)**, subcutaneous adipose tissue (SAT) area **(B)** and peri-renal adipose tissue (RAT) volume **(C)**, SAT area **(D)**, VAT area **(E)**, and RAT volume **(F)**. r: Pearson's simple regression analysis, p: *p*-values.

We finally explored the relationship between markers of renal function (eGFR) and adiponectin in plasma and SAT and RAT (Figure 2). SAT adiponectin, but not plasma adiponectin and RAT adiponectin, was positively correlated with eGFR. VAT area and RAT volume, but not SAT area, were negatively correlated with eGFR.

## Discussion

Current study evaluated determinants of plasma adiponectin levels and expression levels of adiponectin in SAT and RAT in human samples. We found that: first, plasma adiponectin levels were correlated with VAT area, but not with BMI, waist circumference, SAT area and RAT volume; second, expression levels of adiponectin in SAT were correlated with BMI, waist circumference, and SAT area, but not with VAT area and RAT volume; and third, expression levels of adiponectin in RAT were correlated with all adiposity indices including BMI, waist circumference, SAT area, VAT area, and RAT volume. Determinants of plasma adiponectin and adiponectin in SAT were the same as previous reports (1–5). Determinants of adiponectin in RAT were first evaluated in the current study, and then now we found that those were correlated well with remote fat mass such as in SAT and VAT and also with local fat mas as in RAT.

### Determinants of Plasma Adiponectin

Current study found that determinant of plasma adiponectin was exclusively VAT in agreement with previous studies (2–4). The fact indicates that the circulating level of adiponectin is, paradoxically, decreased with increasing central obesity and visceral fat obesity (2–4), although adiponectin is secreted by the adipose tissue (2–4). Therefore, it is considered that the location of fat depots differentially influences circulating adiponectin concentrations. Adiponectin is a stable protein in the circulation with minimal degradation observed while circulating (25), but it has a short plasma half-life of ~45–75 min, depending on the complex and the conditions (26). It was believed that the liver is the primary site of adiponectin clearance from the circulation (26), but other cell types especially in the cardiovascular system and kidney may be involved in the clearance of plasma adiponectin through adiponectin/T-cadherin system (27). Taken together, the link between plasma adiponectin and visceral fat mass can be related to the production and/or clearance of plasma adiponectin.

### Determinants of Adiponectin in Subcutaneous Adipose Tissue

Expression levels of adiponectin in SAT were correlated with BMI, waist circumference, and SAT area, but not with VAT area and RAT volume. Previous studies have reported a negative association between adiponectin expression in SAT and BMI, waist circumference and SAT mass (10, 28). Perrini et al. reported that mRNA expression levels of adiponectin were higher in SAT than in VAT. In contrast, cultured visceral adipocytes exhibited a higher release than subcutaneous adipocytes (9). Combined, it is suggested that gene expression and protein secretion of adiponectin may be regulated differently in a fat depots-specific manner. Indeed, gene expression is under the transcriptional control of C/EBPα (29), which was found to be expressed at higher levels in SAT than in VAT (10), whereas secretion can be regulated by other factors including insulin (30). Also, it was demonstrated that Sirt1, a nicotinamide adenosine dinucleotide-dependent deacetylase, stimulates various gene transcription by cooperating with forkhead box O1 (FOXO1) and C/EBPα (31) but also suppresses secretion (32). Thus, it is hypothesized that SIRT1 could be differently expressed and/or activated in VAT than in SAT (10). In the present study, SAT adiponectin levels were negatively with CD68 and NOX4. Also, SAT area and RAT volume were positively correlated with CD68 levels. The negative correlation between SAT adiponectin and SAT pro-inflammatory markers may suggest an association between decreased SAT adiponectin and increased local inflammation.

### Determinants of Adiponectin in Peri-Renal Adipose Tissue

Previous studies suggest that excessive peri-renal fat increases the risk of cardiometabolic risk (18), hypertension (19), and CVD (20). Therefore, it is suggested that peri-renal fat is associated with kidney dysfunction and/or cardiometabolic risk via paracrine signaling of adipocytokine (17). To our best knowledge, we, for the first time, measured the link between peri-renal fat mass and the region-specific adiponectin expression. Our results showed that expression levels of adiponectin in RAT were correlated well with remote fat mass such as in SAT and VAT and also with the local peri-renal fat mass. RAT volume was positively correlated with CD68 levels and RAT adiponectin levels were negatively correlated with NLRP3, TLR2, and CD68. As in SAT, this negative correlation between RAT adiponectin and RAT pro-inflammatory markers would suggest an association between decreased adiponectin and increased local inflammation in local RAT.

Accumulation of RAT is assumed to be linked to kidney dysfunction (16, 17). SAT adiponectin, but not plasma adiponectin and RAT adiponectin, was positively correlated with eGFR. Meanwhile, VAT area and RAT volume, but not SAT area, were negatively well correlated with eGFR. Although RAT adiponectin was not significantly correlated with kidney function, a decrease in RAT adiponectin may be linked to kidney dysfunction at least partly through an increase in RAT volume. Our study may also imply that accumulations in peril-renal fat are correlated with a decrease in adiponectin and an increase in proinflammatory adipocytokines (Table 3). Combined all above, it might be assumed that peri-renal fat belongs to intra-abdominal fat such as VAT and has a unique characteristic which may locally or directly contribute to kidney function through local chronic inflammation.

## Limitations

There are limitations in this study. First, the present study entirely recruited Japanese patients, and the number of patients is rather small. Second, the studied population was heterogenous in terms of metabolic backgrounds including age, gender, adiposity, and glucose intolerance. Also, we cannot exclude influence of underlying diseases, mainly cancer or tumors, on the gene expression in adipose tissues. Collectively, our data cannot represent the general population. Especially, effects of RAT volume and RAT adiponectin should be explored in non-cancer participants in future studies. Third, we could not measure levels of adiponectin protein in renal biopsied samples. Adiponectin is an adipocyte-derived 30 kDa secretory protein, and monomeric adiponectin is posttranslationally modified and oligomerized to form trimers (low molecular weight, LMW), hexamers (medium, MMW), and biologically active oligomeric (high, HMW) forms (8, 33). Thus, expression levels of adiponectin are not necessarily linked to its biological function of HMW adiponectin. We could measure only mRNA but not protein levels of adiponectin. The relationship between secretion of HMW, MMW, and LMW forms and kidney functions might be warranted in future studies. Last, we could not measure other obesity-associated adipokines such as other obesity-associated adipocytokines such as TNFα, leptin, IL6, IL8, MCP1, and FABP4 because of the limited quantity of samples.

## Conclusion

**Figure 3:**
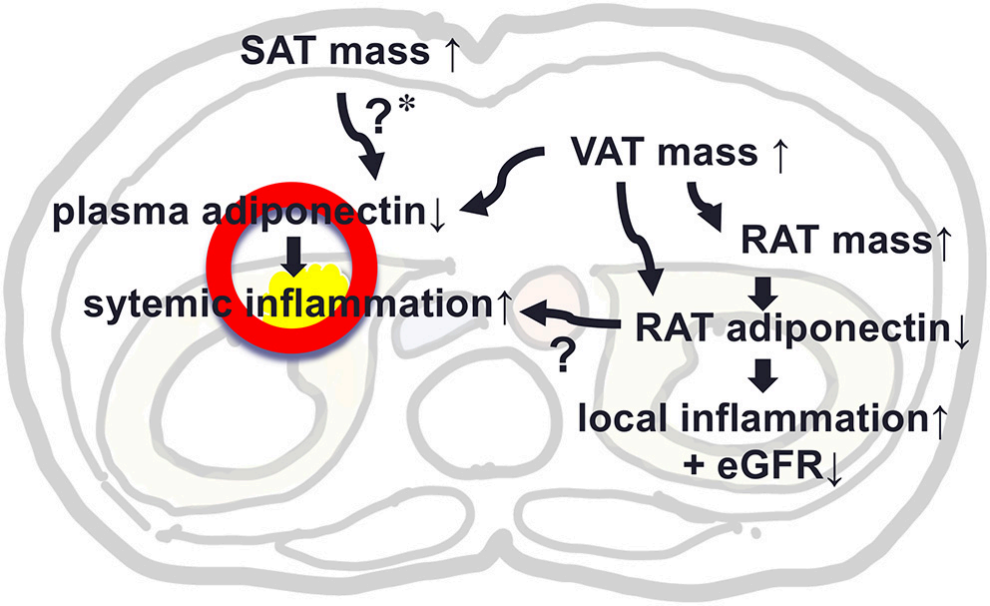
A key Illustration explaining the possible role of adiponectin in peri-renal adipose tissue (RAT) and RAT volume in kidney function and local and systemic inflammation. *In our study, SAT mass was not associated with plasma level of adiponectin, but previous studies support that SAT mass was positively associated with plasma adiponectin, suggesting a protective effect against hypoadiponectinemia by increased VAT mass.

This study evaluated levels of adiponectin expression in RAT and its determinants as compared to that of SAT in patients who underwent urological operation. Levels of adiponectin mRNA in RAT were negatively correlated with remote fat mass in SAT and VAT and also with local fat mass in RAT, while, level of adiponectin in SAT was not correlated with RAT volume. We made a key Illustration explaining the possible role of RAT adiponectin and RAT volume in kidney function and local and systemic inflammation (Figure 3). Further studies are warranted to evaluate the roles of peri-renal fat mass accumulation and its pathophysiological machineries.

## Data Availability Statement

The raw data supporting the conclusions of this article will be made available by the authors, without undue reservation, to any qualified researcher.

## Ethics Statement

The studies involving human participants were reviewed and approved by the institutional review boards of the University of Tokushima Hospital. The patients/participants provided their written informed consent to participate in this study.

## Author Contributions

GM and MSh take responsibility for all aspects of the reliability and freedom from bias of the data presented and their discussed interpretation. HI, YH, and HK performed sampling and patients management. DF, HM, and MSa discussed data, reviewed the manuscript, and approved the final manuscript.

### Conflict of Interest

The authors declare that the research was conducted in the absence of any commercial or financial relationships that could be construed as a potential conflict of interest.
